# Supplementary material for: Molecular Epidemiology and Antibiotic Resistance Associated with Avian Pathogenic Escherichia coli in Shanxi Province, China, from 2021 to 2023
Source: Microorganisms. 2025 Feb 27;13(3):541. doi: 10.3390/microorganisms13030541 (PMC11946381; doi:10.3390/microorganisms13030541)
Supplement: Supplementary file 1 [file microorganisms-13-00541-s001.zip › microorganisms-3471516-supplementary.pdf]

Supplementary Materials: The following supporting information can be downloaded at: https://www.mdpi.com/article/10.3390/microorganisms13030541/s1.

Figure S1: Dichotomous decision tree to determine the phylogenetic group of an *E. coli* strain by using the results of PCR amplification of the genes *chuA*, *yiaA*, and *TspE4.C2*.

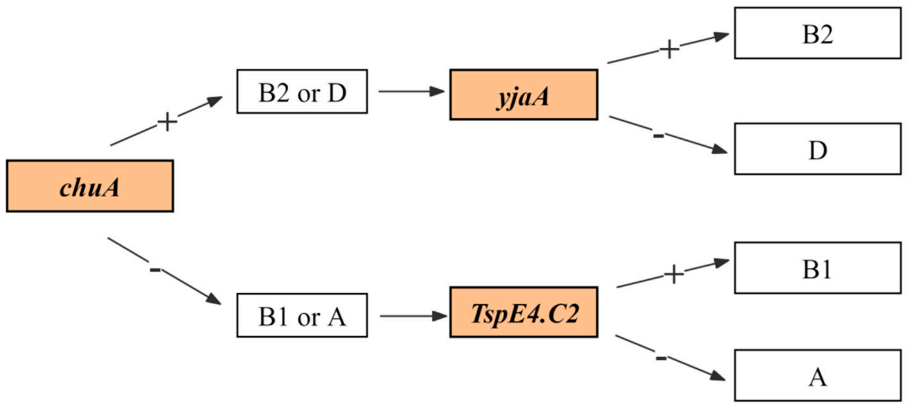

Table S1: Primers used for detection of common resistance genes of *Escherichia coli.*

Types of resistance genes	Genes	Primer	Sequence (5'→3')	Product length / bp
Tetracyclines	*tetA*	tetA-F	GCTACATCCTGCTTGCCTTC	210
		tetA-R	CATAGATCGCCGTGAAGAGG	
β-lactams	*bla*-CTX M	CTX-M-F	AACCGTCACGCTGTTGTTAG	766
		CTX-M-R	TTGAGGCTGGGTGAAGTAAG	
	bla-TE M	TEM-F	GAGTATTCAACATTTTCGT	857
		TEM-R	ACCAATGCTTAATCAGTGA	
Amphenicols	*cat1*	cat1-F	AGTTGCTCAATGTACCTATAACC	547
		cat1-R	TTGTAATTCATTAAGCATTCTGCC	
	floR	floR-F	CGCCGTCATTCCTCACCTTC	215
		floR-R	GATCACGGGCCACGCTGTGTC	
Sulfonamides	*sul1*	sul1-F	CGGCGTGGGCTACCTGAACG	433
		sul1-R	GCCGATCGCGTGAAGTTCCG	
	sul2	sul2-F	GCGCTCAAGGCAGATGGCATT	293
		sul2-R	GCGTTTGATACCGGCACCCGT	
Aminoglycosides	*strA*	strA-F	ATGGTGGACCCTAAAACTCT	891
		strA-R	CGTCTAGGATCGAGACAAAG	
	aphA	aphA-F	ATGGGCTCGCGATAATGTC	603
		aphA-R	CTCACCGAGGCAGTTCCAT	
Aminoglycosides	*qnrA*	qnrA-F	AGAGGATTTCTCACGCCAGG	580
		qnrA-R	TGCCAGGCACAGATCTTGAC	
polypeptide	*mcr 1*	mcr 1-F	CGGTCAGTCCGTTTGTTC	309
		mcr 1-R	CTTGGTCGGTCTGTAGGG	

Table S2: Primers used for detection of virulence genes of *Escherichia coli.*

Types of virulence genes	Genes	Primer	Sequence (5'→3')	Product length / bp
adhesin-related genes	*aatA*	aatA-F	CTACACTCCGATTCCTCTGG	713
		aatA-R	CATCCGTTCTGGCACTATTT	
	papC	papC-F	GCACCATTTGTATAATCTGCGCCC	627
		papC-R	TGAGAGATGATCGTTCCGTCAGGA	
	tsh	tsh-F	GCAGAAAATTCAATTTATCCTTGG	537
		tsh-R	CTGATAAGCGATGGTGAATTAAC	
	fimC	fimC-F	TATGTTGGCTTTGAAATGGG	513
		fimC-R	ATCCAGAGCAGCCTGACCTT	
	mat	mat-F	GCAAAGTATTTCTCCCGCATC	1185
		mat-R	CGGCATCCGCATTATCAAAC	
	vat	vat-F	GTCATTGGACCTGATGGATA	177
		vat-R	GACTTCATTTGAGCGTGTTG	
iron transport-related factors	*fyua*	fyua-F	GGCGGCGTGCGCTTCTCGCA	209
		fyua-R	CGCAGTAGGCACGATGTTGTA	
	irp2	irp2-F	TGATTTCTGGCGCACCATCT	794
		irp2-R	GTCATGTTCGGCCAGGATG	
	iucD	iucD-F	TCAGTCATACCGTTGAAAAC	482
		iucD-R	CAACTCACGATAAATGGTCA	
invasion-related genes	*ibeB*	ibeB-F	GCGTGTTGCTGAAGTGAGTG	585
		ibeB-R	TTGCCAGTTCCAAATGTTCT	
	yijP	yijP-F	ATGATTGTGACTGGCTAACG	260
		yijP-R	TTGGCAAAGATACCACTTCT	
	ibeA	ibeA-F	GTATGACGGTGGGAACAAGAG	321
		ibeA-R	TGGCAATAGCAGCGGCAGTC	
serum resistance factor-related genes	*ompA*	ompA-F	ATGATGGTCATCCGTCCCGT	620
		ompA-R	ATCAGTTCTGCAATAAATGC	
	neuC	neuC-F	AAGAACTTGAAGAGGGAGACT	774
		neuC-R	CACCGACACTAATGATGACC	
	cva	cva-F	TGCCGTGGTTTAGTCGGATTG	456
		cva-R	TGCGATTTAAGGCGTTCATG	
	iss	iss-F	TGTCAGGACGGCAGATGAAC	1491
		iss-R	TTTCCGGACTGCCTCCTTTC	
	iroN	iroN-F	GTATACGATGACGCCGGGAG	236
		iroN-R	GCCTGTCGCCAGTTATCTGA	
